# Overexpression of TGF-*β* by infiltrated granulocytes correlates with the expression of collagen mRNA in pancreatic cancer

**DOI:** 10.1038/sj.bjc.6602141

**Published:** 2004-09-07

**Authors:** Y Aoyagi, T Oda, T Kinoshita, C Nakahashi, T Hasebe, N Ohkohchi, A Ochiai

**Affiliations:** 1 Pathology Division, National Cancer Center Research Institute East, Kashiwanoha 6-5-1, Kashiwa, Chiba 277-8577, Japan; 2 Hepato-Biliary-Pancreatic Surgery Division, National Cancer Center Hospital East, Kashiwanoha 6-5-1, Kashiwa, Chiba 277-8577, Japan; 3 Department of Surgery, Institute of Clinical Medicine, University of Tsukuba, Tennodai 1-1-1, Tsukuba, Ibaraki 305-8575, Japan

**Keywords:** pancreatic cancer, desmoplastic reaction, TGF-*β*, granulocytes, quantitative RT–PCR

## Abstract

Pancreatic cancer is often associated with an intense production of interstitial collagens, known as the desmoplastic reaction. To understand more about desmoplasia in pancreatic cancer, the expression of mRNA for type I and III collagens and potent desmoplastic inducing growth factors transforming growth factor-*β* (TGF-*β*), connective tissue growth factor (CTGF), acidic and basic fibroblast growth factor (FGF), platelet-derived growth factor (PDGF) A and C and epidermal growth factor (EGF) was analysed by quantitative RT–PCR. Expression of both collagens in 23 frozen primary pancreatic cancer nodules was significantly higher than that in 15 non-neoplastic pancreatic tissues. The expressions of mRNAs for TGF-*β*, acidic FGF, basic FGF and PDGF C were likewise higher in surgical cancer nodules, while that of CTGF, PDGF A and EGF were not. Among these growth factors, the expression of TGF-*β* mRNA showed the most significant correlation with that of collagens (*P*<0.0001). By immunohistochemistry, TGF-*β* showed faint cytoplasmic staining in cancer cells. In contrast, isolated cells, mainly located on the invasive front surrounding cancerous nests, were prominently and strongly stained. These TGF-*β*-positive cells contained a segmented nucleus, were negative for anti-macrophage (CD68) and positive for anti-granulocyte antibodies, indicating their granulocytic nature. In conclusion, TGF-*β* seemed to play a major role among the various growth factors in characteristic overproduction of collagens in pancreatic cancer. Moreover, the predominant cells that express TGF-*β* were likely to be infiltrated granulocytes (mostly are neutrophils) and not pancreatic cancer cells.

Pancreatic adenocarcinoma is a highly lethal disease with no definitive therapy (Warshaw and Fernandez-del Castillo, 1992; Neoptolemos *et al*, 2003). One hallmark of pancreatic adenocarcinoma is the intense production of interstitial components, known as the desmoplastic reaction (Iacobuzio-Donahue *et al*, 2002). As a result, the proportion of pancreatic cancer cells is less than 20 – 40% of the tumour mass (Kloppel *et al*, 1985), while the remaining 60 – 80% includes interstitial cells (fibroblast, endocells and inflammatory cells) and proliferated interstitial components include collagens, followed by fibronectin, laminin and proteoglycan (Gress *et al*, 1995; Imamura *et al*, 1995; Linder *et al*, 2001).

The desmoplastic reaction has been reported to be stimulated by various growth factors including epidermal growth factor (EGF) (Gospodarowicz, 1983), platelet-derived growth factor (PDGF) (Gospodarowicz, 1983; Tahara, 1990), connective tissue growth factor (CTGF) (Frazier *et al*, 1996), fibroblast growth factor (FGF) (Gospodarowicz, 1983; Powers *et al*, 2000) and transforming growth factor-*β* (TGF-*β*) (Roberts *et al*, 1986; Fine and Goldstein, 1987; Sappino *et al*, 1990; McCartney-Francis and Wahl, 1994). In various malignancies including pancreatic cancer, overexpression of these growth factors is frequently observed (Korc *et al*, 1992; Friess *et al*, 1993; Yamanaka *et al*, 1993; Ebert *et al*, 1995; Gold, 1999; Wenger *et al*, 1999) and has been associated with a significant decrease in the survival and advanced tumour stage (Friess *et al*, 1993). Comprehensive analysis, however, concerning the growth factors that have the strongest impact on the induction of the desmoplastic reaction has never been reported; furthermore, as to which one of the cellular component (i.e. neoplastic cells and interstitial cells) contributing to the overexpression of growth factors inducing desmoplasia in pancreatic cancer has remained obscure. It is generally believed that the cancerous component secretes the growth factors that give rise to this host reaction (Korc *et al*, 1992; Friess *et al*, 1993; Yamanaka *et al*, 1993; Ebert *et al*, 1995; Gold, 1999; Wenger *et al*, 1999). However, as haematopoietic cells including lymphocytes, macrophages and granulocytes are also capable of secreting growth factors (Roberts and Sporn, 1988; Grotendorst *et al*, 1989; Leonardi *et al*, 2000), we hypothesised that infiltrated haematopoietic cells in addition to cancer cell itself could be a source of growth factors that result in induction of desmoplasia.

In order to better understand the molecular mechanism of behind desmoplasia in pancreatic cancer, we have analysed the expression of mRNA of collagens and potent desmoplastic inducing growth factors. We demonstrate that expression of collagens was significantly correlated with that of TGF-*β* in primary pancreatic cancers, and, moreover, that the main source of TGF-*β* is likely to be infiltrated granulocytes (mostly are neutrophils) and not cancer cells.

## MATERIAL AND METHODS

### Surgical resected tissues of human pancreatic cancer

The pancreatic cancer tissues used in this study were obtained from patients (10 male; 13 female) undergoing surgery for pancreatic adenocarcinoma in the National Cancer Center Hospital East Japan from 1999 to 2002. The median age was 66 years, ranging from 52 to 81. There was one patient with stage I, two patients with stage II, 10 patients with stage III and 10 patients with stage IV disease. In all, 15 non-neoplastic pancreatic tissues obtained from the same patients were also evaluated. Specimens ranging from 100 to 300 mg were immediately homogenised in TRIZOL reagent solution (Life Technologies, Gaithersburg, MD, USA) using multi-beads shocker (YASUI kikai, Osaka, Japan) after surgical removal. Samples were stored at −80°C until RNA was extracted.

### Cultured cell lines

Six human pancreatic cancer cell lines were analysed. ASPC-1, BxPC-3, CAPAN-1 and MiaPaca-2 were obtained from the American Type Culture Collection (ATCC) (Bethesda, MD, USA), PSN-1 was from the Central Animal Laboratory National Cancer Center Research Institute (Tokyo, Japan) and SUIT-2 cells were generously provided by Dr Iwamura (Miyazaki Medical College, Miyazaki, Japan). Two gastric cancer cell lines (KATO3 and MKN45), two colon cancer cell lines (COLO201 and SW1116) and two fibroblast (MRC-5 and WI-38) cell lines were also analysed (ATCC). All cell lines were grown in either RPMI1640 or Dulbecco's modified Eagle medium (Sigma Aldrich, Taufkirchen, Germany) containing 10% heat-inactivated foetal bovine serum (Sigma). All cell lines were kept in a humidified atmosphere containing 5% CO_2_ at 37°C. Approximately 1 × 10^7^ cells were sheared in 1 ml of TRIZOL reagent solution using a 21G needle. The homogenate was kept at −80°C until RNA was extracted.

### RNA extraction

RNA from surgically resected tissues was extracted from about 100 mg of homogenised tissue in TRIZOL reagent solution. Samples were treated with 40 U of RNase-free DNase I (TAKARA, Shiga, Japan) in 200 *μ*l DEPC (diethylpyrocarbonate)-treated water, 10mM MgCl_2_ and 40 U of RNase Inhibitor (TOYOBO, Osaka, Japan) at room temperature for 15 min.

### Reverse transcription (RT)

All cDNAs were synthesised from 10 *μ*g total RNA using 50 *μ*M oligo (dT)_20_ primer in a total volume of 50 *μ*l using ThermoScript™ RT–PCR System (Life Technologies) according to the manufacturer's protocol. cDNAs were purified using the QIA quick PCR purification Kit (QIAGEN, Hilden, Germany) and eluted in 100 *μ*l of 10 mM Tris–HCl (pH 8.5).

### Real-time quantitative RT – polymerase chain reaction (RT – PCR)

Expression of type I collagen and type III collagen mRNAs was analysed since they are major proteins of the stromal component. Transforming growth factor-*β*, CTGF, acidic FGF (aFGF), basic FGF (bFGF), PDGF A, PDGF C and EGF were analysed as they are potent desmoplastic inducing molecules. Polymerase chain reaction primer pairs for mRNA quantification, intending to flank at least one intron, were designed based on coding sequences obtained from the GenBank Sequence Database (http://www.ncbi.nlm.nih.gov/Ge nbank/index.html) (Table 1

**Table 1:** PCR primers for collagens and potent desmoplastic inducing growth factors

**Gene**	**Accession no.**	**Position**	**Product length (bp)**	**Forward primer sequence**	**Reverse primer sequence**
Type I collagen	NM_000088	4010–4390	381	5′-CAG TTC GAG TAT GGC GGC G-3′	5′-GAC AGG GCC AAC GTC GAA G-3′
Type III collagen	NM_000090	3632–3936	305	5′-AAA AAG CTG GCG GTT TTG CC-3′	5′-CCG TGG AAC ATT CAA AGG AT-3′
TGF-*β*	X02812	402–651	250	5′-CTT CAA CAC ATC AGA GCT CCG A-3′	5′-GCC CTC AAT TTC CCC TCC AC-3′
CTGF	M92934	503–871	369	5′-AGC CCA AGG ACC AAA CCG TG-3′	5′-GGC CGT CGG TAC ATA CTC CA-3′
aFGF	X65778	318–468	150	5′-GAA CCA TTA CAA CAC CTA TAT-3′	5′-TTA ATC AGA AGA GAC TGG C-3′
bFGF	M27968	254–445	192	5′-TGA AGG AAG ATG GAA GAT TA-3′	5′-GAA AAA GTA TAG CTT TCT GC -3′
PDGF A	A09204	266–538	273	5′-AGG AAG CTG TCC CCG CTG TC-3′	5′-CGC AGG CGC ACT CCA AAT GC-3′
PDGF C	AF260738	493–739	247	5′-CCA CAA TTC ACA GAA GCT GT-3′	5′-ATC TTA CCT CCT CTG TTA GAA GG-3′
EGF	NM_001963	3246–3586	341	5′-GCC TGC TGA CAC TGA GGA T-3′	5′-CCC TTT GTT GCC ATA ATG GG-3′

).

### Quantitative real-time RT–PCR

Quantitative real-time PCR was carried out using a LightCycler™ instrument (Roche, Mannheim, Germany) using SYBR green. In all, 1 *μ*l of cDNA solution corresponding to 100 ng of total RNA was subjected to 40 PCR cycles of 10 s at 95°C, 10 s at 53 – 65°C and 5 – 15 s at 72°C in a 10 *μ*l mixture containing 1 *μ*l LightCycler-DNA Master SYBR Green I (Roche), 2.25 – 5 mM MgCl_2_ and 0.25 *μ*M each of forward and reverse gene-specific primers. Polymerase chain reaction conditions and detection temperature of fluorescent products were optimised for each gene by meticulous pilot studies (Table 2

**Table 2:** PCR conditions

**Gene**	**MgCl_2_ (mM)**	**Annealing temp. (°C)**	**Elongation time (s)**	**Detect temp. (°C)^a^**
Type I collagen	2.25	63	15	88
Type III collagen	4	63	10	80
TGF-*β*	3	65	8	86
CTGF	2.25	66	13	85
aFGF	4	53	5	81
bFGF	5	58	7	72
PDGF A	3	68	10	88
PDGF C	3	56	9	79
EGF	4	65	13	72

a Temperature for detection of fluorescent PCR products.

). Amplification specificities of the PCR products were confirmed by melting curve analysis of the LightCycler instruments, and re-confirmed by agarose gel electrophoresis.

An external standard curve for each gene was generated using serial 10^2^-fold dilutions of RT–PCR products, corresponding to 1 × 10^8^ – 1 × 10^2^ copies *μ*l^−1^, to estimate the gene-specific mRNA copy number per 100 ng total RNA of each sample.

### Immunohistochemistry

The expression of TGF-*β* was investigated by immunohistochemistry (IHC) using anti-human TGF-*β*1 antibody in 23 pancreatic cancer tissues. Potent TGF-*β*-producing haematopoietic cells, that is, macrophages or granulocytes, were also visualised by IHC. Paraffin-embedded, formalin-fixed sections were subjected to antigen retrieval by immersion in 0.1 M citrate buffer (pH 6.0) and microwaving at 95°C or by incubation with protease K (DAKO, Glostrup, Denmark) at room temperature for 10 min. Endogenous peroxidase was inactivated by incubating in 0.3% H_2_O_2_ in methanol for 10 – 20 min. Nonspecific binding was blocked by treatment with 5% skim milk and 2% bovine serum albumin in PBS for 30 min. Tissues were then incubated with polyclonal rabbit antibodies against anti-human TGF-*β*1 (Santa Cruz Biotechnology, Inc., Santa Cruz, CA, USA), as well as anti-human CD68 mouse monoclonal antibodies (DAKO), and anti-human granulocyte (Medical & Biological Laboratories Co., Nagoya, Japan) at a dilution of 1 : 100 in a humidified chamber at 4°C overnight. Primary antibody reactions of TGF-*β* and granulocyte were enhanced using the Envision+kit (DAKO). CD68 antibody treatment was followed by incubation with rabbit anti-mouse secondary antibody and enhanced using Strept AB Complex/HRP kit (DAKO). The immunoreaction was visualised with 0.05% 3,3′-diaminobenzidine (DAB) solution for 1 – 10 min at room temperature. After washing in distilled water, the specimens were counterstained with haematoxylin, dehydrated and mounted.

As negative control for TGF-*β*1 analysis, the primary antibody was substituted for by anti-green fluorescence protein (GFP) rabbit polyclonal antibodies (Molecular Probes, Inc., Eugene, OR, USA) and processed as described above. As a negative control for granulocyte and macrophage visualisation, anti-mouse H2Kb mouse monoclonal antibodies (PharMingen, San Diego, CA, USA) were used.

### Double immunofluorescence stain

In order to corroborate the precise localisation of TGF-*β*, five representative pancreatic cancer tissues were subjected to double immunofluorescence staining. Sections were incubated with the primary antibody pairs against (TGF-*β*+CD68) or (TGF-*β*+granulocyte) for 1 h at room temperature in a humidified chamber. Transforming growth factor-*β* was labelled red with Alexa Fluor 546 F(ab′)_2_ fragments of goat anti-rabbit IgG (Molecular Probes, Inc., OR, USA) at a dilution of 1 : 1000, and CD68 and granulocytes were labelled green with fluorescein (FITC) horse anti-mouse IgG (Vector Laboratories, Inc., CA, USA) at a dilution of 1 : 100 by incubation for 30 min at room temperature. The sections were mounted in PermaFlior™ Aqueous Mounting Medium (ThermoShandon, PA, USA) and examined with a MRC-1024 confocal imaging system (BIO-RAD, Herts, UK).

### Statistical analysis

As the expression of mRNAs for type I collagen, type III collagen, TGF-*β*, CTGF, aFGF, bFGF, PDGF A, PDGF C and EGF exhibited asymmetrical distributions, nonparametric tests (Wilcoxon paired tests) were used. The relationship between expression of collagens and TGF-*β*, CTGF, aFGF, bFGF, PDGF A, PDGF C and EGF was assessed by linear regression analysis (Mann–Whitney *U* test). Significance was defined as *P*<0.05. Statistical calculations were performed with the Stat View software package (Version 5.0: Abacus Concepts, Inc., Berkeley, CA, USA).

## RESULTS

### Expression of mRNAs for collagens and growth factors in surgical specimens

mRNA copy numbers are listed in Table 3

**Table 3:** Expression of collagens and potent desmoplastic inducing growth factors

**Gene**	**Copy number × 10^2^ copies/100 ng total RNA**		***P*-value**
Type I collagen	C	2497.1±3322.7	0.018
	N	864.6±1087.4	
			
Type III collagen	C	2103.1±2305.7	0.018
	N	876.0±1143.8	
			
TGF-*β*	C	12.9±15.2	0.026
	N	3.8±3.9	
			
aFGF	C	4.1±5.4	0.002
	N	1.1±2.5	
			
bFGF	C	4.6±6.5	0.034
	N	1.8±2.2	
			
PDGF C	C	14.9±23.7	0.028
	N	5.3±11.0	
			
CTGF	C	212.8±380.3	0.170
	N	96.9±159.7	
			
PDGF A	C	3.4±3.1	0.347
	N	3.9±5.3	
			
EGF	C	1.3±3.1	0.179
	N	3.1±5.1	
			

**Figure 1:**
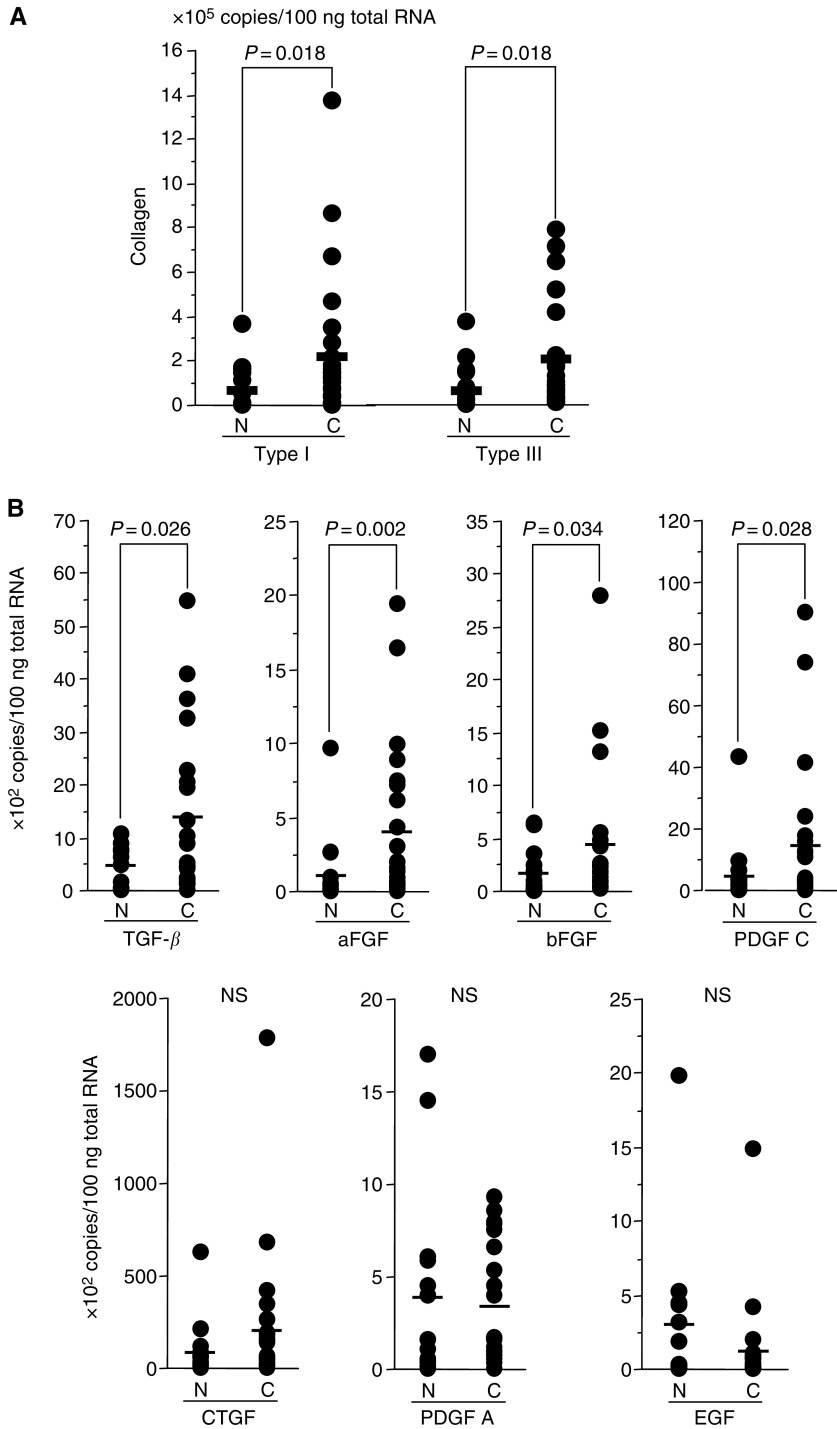
Expressed copy number per 100 ng of total RNA in pancreatic cancerous (C) and noncancerous (N) lesions from surgical specimens as measured by real-time RT–PCR. (**A**) Expressions of type I collagen and type III collagen in C were significantly higher than that of N. (**B**) Expressions of TGF-*β*, aFGF, bFGF and PDGF C in C were significantly higher than that of N, while those of CTGF, PDGF A and EGF were not significant. Bars indicate mean values. NS: not significant.

C=cancerous lesions, N=noncancerous lesions.

and represented in Figure 1

**Figure 2:**
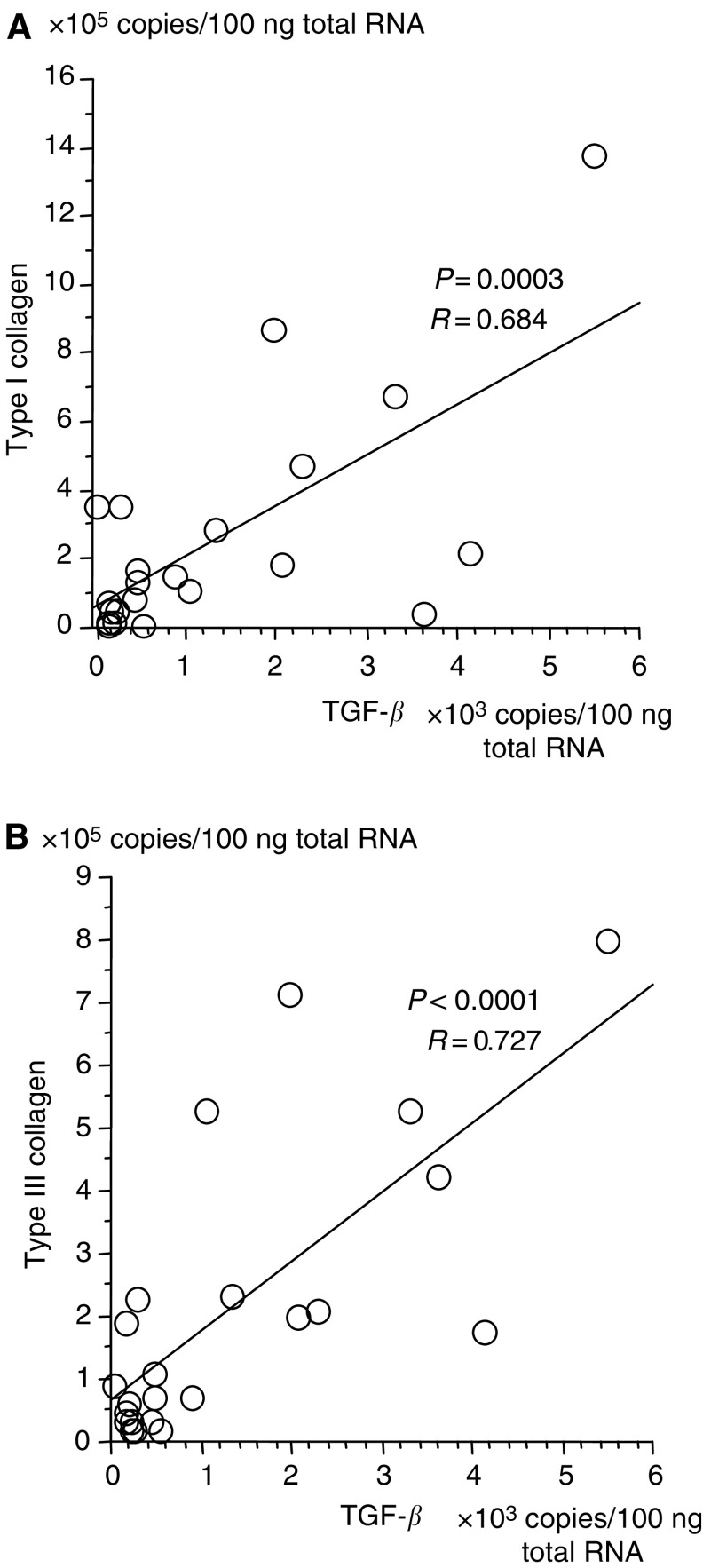
Correlation between TGF-*β* and type I collagen (**A**), and TGF-*β* and type III collagen (**B**) mRNA expression in surgical specimens. The expressed copy number of TGF-*β* and collagens in pancreatic cancer tissues from surgical specimens showed a correlation.

. In surgically resected pancreatic cancerous lesions (=C), the expression of mRNA for type I collagen and type III collagen was significantly higher (2.9- and 2.4-fold, respectively) compared to non-neoplastic pancreatic tissues (=N) (Table 3, Figure 1A). The expression of mRNA for TGF-*β* in C was also higher (3.4-fold) than that in N (Figure 1B). The expression of mRNA for aFGF (3.7-fold), bFGF (2.6-fold), PDGF C (2.8-fold) and CTGF (2.2-fold) was also higher in cancer tissue, while that for PDGF A (−1.1-fold) and EGF (−2.5-fold) was lower (Figure 1B). All growth factors with upregulated expression correlated with type I and type III collagen gene expression. (Type I collagen: TGF-*β* (*r*=0.684, *P*=0.0003), CTGF (*r*=0.436, *P*=0.0376), aFGF (*r*=0.245, *P*=0.2591), bFGF (*r*=0.119, *P*=0.3206), PDGF C (*r*=0.562, *P*=0.0052), Type III collagen: TGF-*β* (*r*=0.727, *P*<0.0001), CTGF (*r*=0.624, *P*=0.0015), aFGF (*r*=0.482, *P*=0.02), bFGF (*r*=0.164, *P*=0.4547), PDGF C (*r*=0.562, *P*=0.0007)). These results indicated that the expression of TGF-*β* showed high correlation with the expression of type I collagen (Figure 2A

) and type III collagen (Figure 2B).

### Type I and type III collagen and TGF-*β* mRNA expression in cell lines

**Figure 3:**
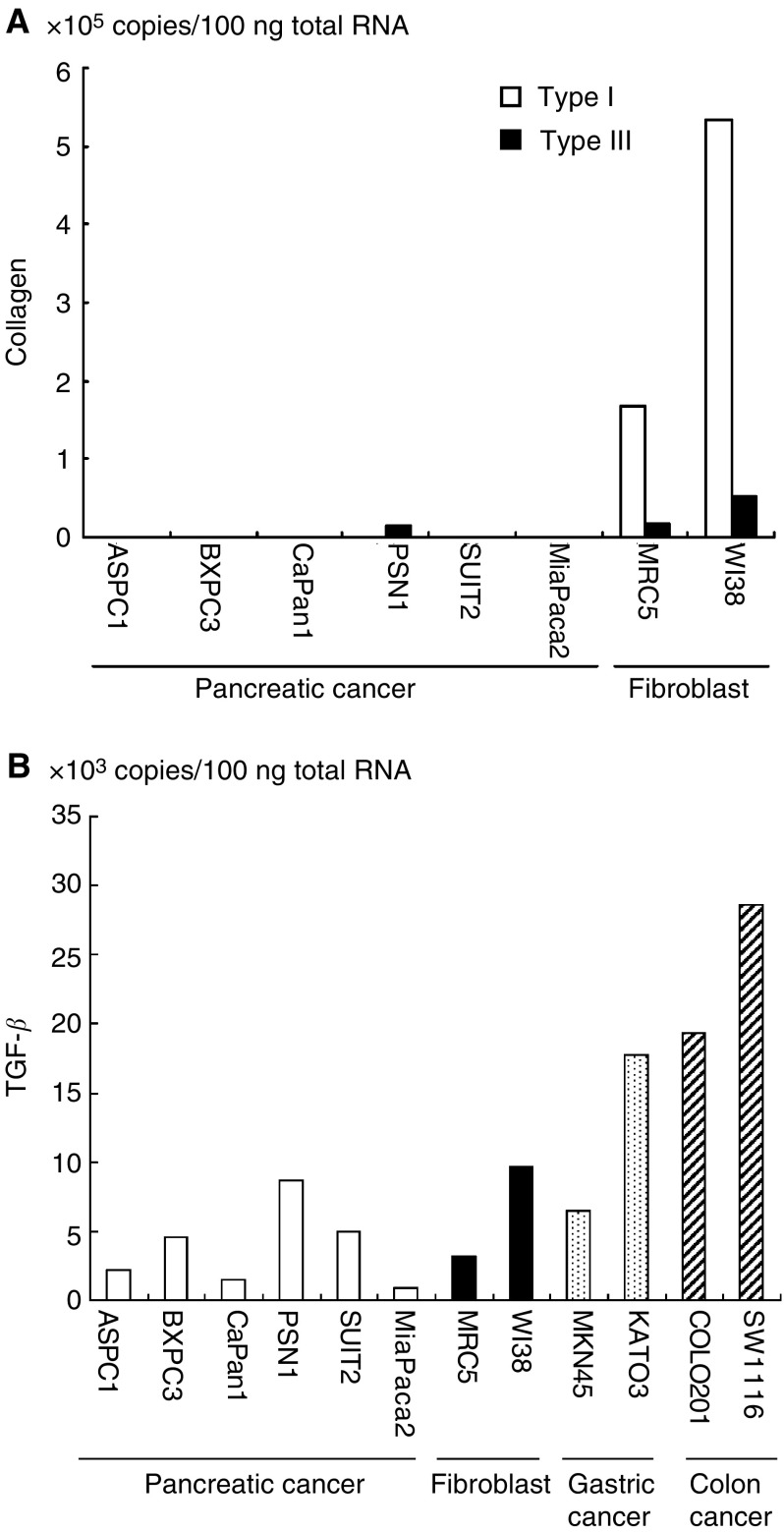
Expression of collagens and TGF-*β* mRNA in various cancer cell lines. (**A**) Expressions of type I and type III collagens were negative in pancreatic cancer cells, except for a small amount of type III in PSN1 compared with fibroblasts. (**B**) Since the expression of TGF-*β* in pancreatic cancer cell lines was the same or less than that in fibroblasts, gastric cancer and colon cancer cell lines, it was presumed that TGF-*β* overexpression is not a specific feature for pancreatic cancer cells.

The copy numbers of the collagens and TGF-*β* mRNA per 100 ng total RNA were analysed for various cell lines. The expression of mRNA for the collagens in fibroblast cell lines was prominent, while the pancreatic cancer cell lines were nearly negative for expression (Figure 3A

). This suggests that fibroblasts may play a crucial role in collagen production, rather than pancreatic cancer cells. Expression of TGF-*β* is not a specific characteristic of pancreatic cancer cell lines and, in fact, cell lines originating from fibroblasts, gastric cancer and colon cancers also express TGF-*β* mRNA at the same or higher levels as the pancreatic cancer cell lines (Figure 3B).

### Immunolocalisation of TGF-*β*

**Figure 4:**
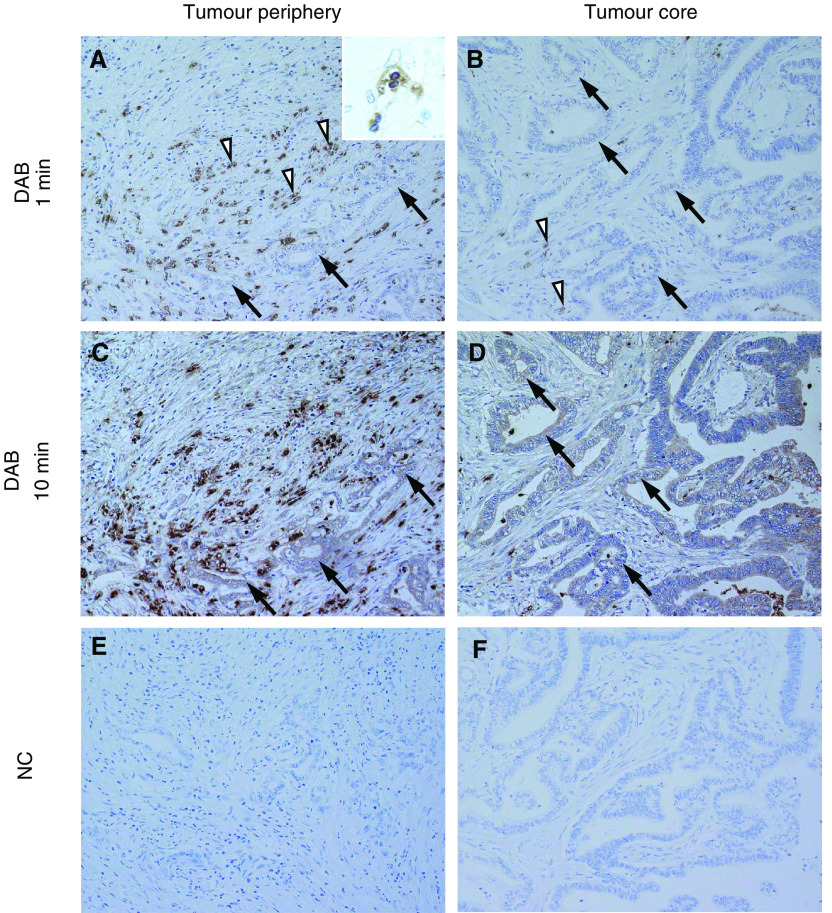
TGF-*β* immunohistochemistry in pancreatic adenocarcinoma. Transforming growth factor-*β* immunostaining was visualised by short (≈1 min) and long (≈10 min) reactions with DAB. Note that staining for cancer cells is barely visible at short DAB staining times (closed arrows) in both the tumour periphery (**A**) and core (**B**), and only slightly apparent after a 10-min reaction (closed arrows) in both the tumour periphery (**C**) and core (**D**). Intense TGF-*β* immunoreactivity was found in granular cells adjacent to the pancreatic cancer nests, even at short DAB incubation periods (open arrow heads) (**A**, **B**). These TGF-*β*-positive cells were predominantly observed at the tumour periphery (**A**), and are rare in the tumour core (**B**). Immunohistochemistry using anti-GFP rabbit polyclonal antibodies as a negative control against anti-TGF-*β* rabbit polyclonal antibody resulted in negative staining in both the tumour periphery (**E**) and core (**F**). NC: negative control.

Since the expression of TGF-*β* mRNA showed a prominent correlation with the expression of collagen mRNA, the protein distribution of TGF-*β* in pancreatic cancer tissues was examined using immunohistochemistry. Immunohistochemistry with TGF-*β* (Figure 4

) demonstrated that the staining in pancreatic cancer cells was extremely faint positive at short DAB reaction times (≈1 min) (arrows in Figures 4A and B), and was barely recognisable after longer incubation (≈10 min) (arrows in Figures 4C and D). In contrast, highly prominent immunostaining was observed in isolated cells bordering the cancer nests even at short DAB reaction times (arrow heads in Figures 4A and B). These TGF-*β*-positive cells were predominantly distributed at the invasive front area (or tumour periphery) (Figures 4A and C), but were rare in the tumour core (Figures 4B and D). High-power field observation of these cells revealed that they possessed bandform and/or segmented nuclei and were presumed to be haematopoietic granulocytes (Figures 4A and C). Immunohistochemistry with anti-GFP rabbit polyclonal antibodies demonstrated complete negative staining for these isolated cells (Figures 4E and F), demonstrating that immunostaining with anti-TGF-*β* was not an artefact but a result of true immuno-reaction between antigens. The other stromal components such as fibroblasts and endothelial cells showed only weak or no immunostaining (Figure 4).

**Figure 5:**
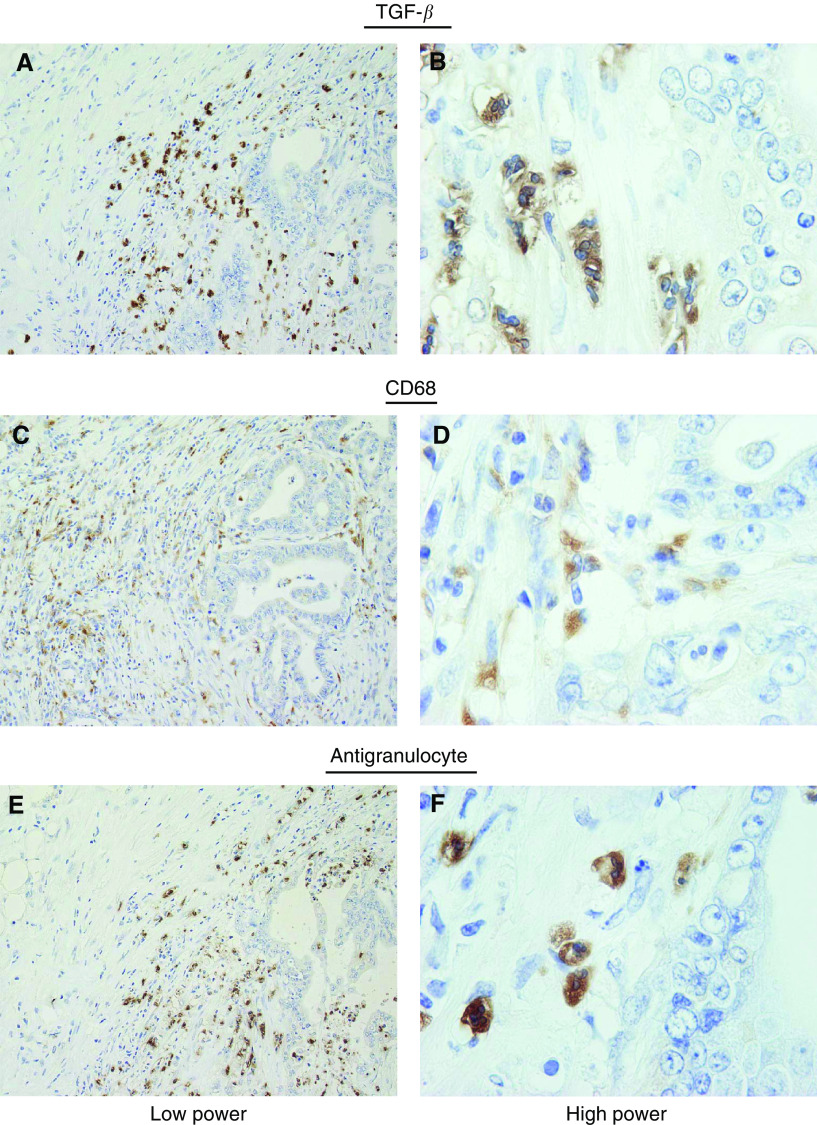
Distribution of TGF-*β*, CD68 and antigranulocyte-positive cells in pancreatic adenocarcinoma. The distribution of isolated TGF-*β*-positive cells in pancreatic cancer (**A**) was similar to that of macrophage (i.e. CD68+ cells) (**C**) and granulocytes (**E**) in low power field observation. However, in high power field observation, the morphology of TGF-*β*1-positive isolated cells (**B**) coincided with antigranulocyte-positive segmented nucleus cells (**F**) but not CD68+ mononuclear cells (**D**).

**Figure 6:**
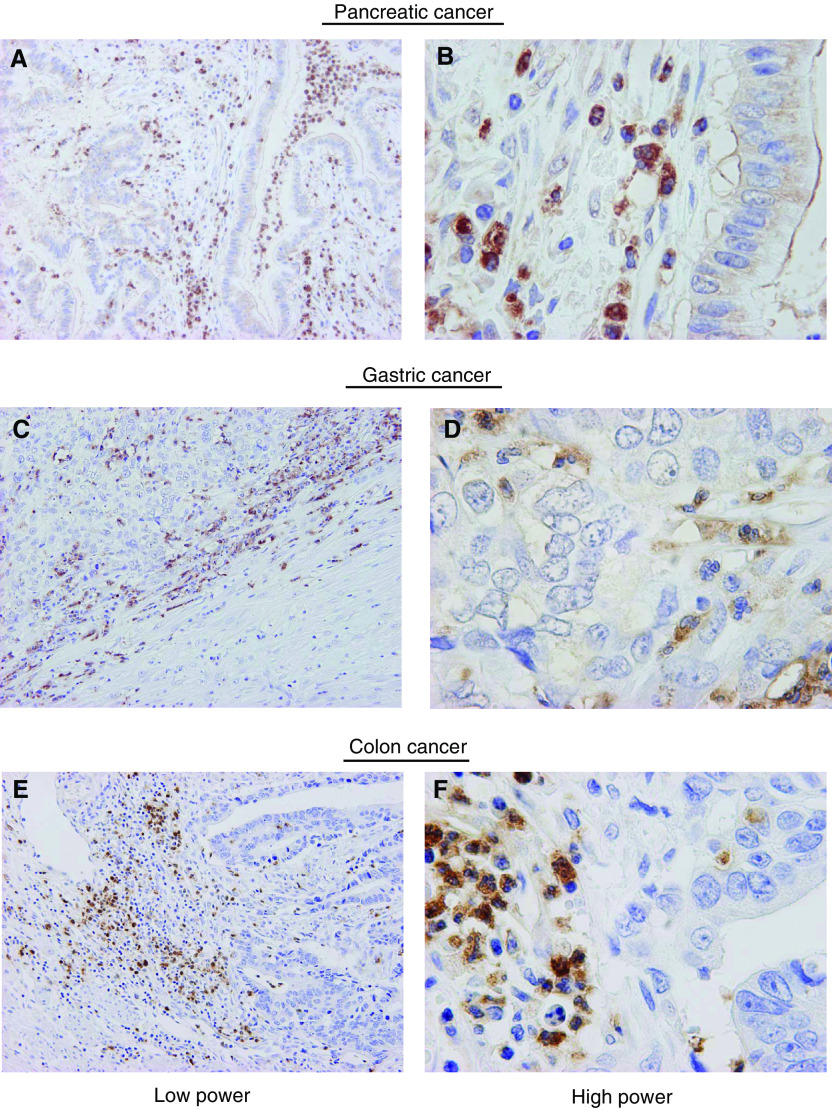
TGF-*β* immunoreactivity in pancreatic, gastric, and colon cancer tissues. TGF-*β* immunoreactivity was found in isolated cells around cancer nests in pancreatic cancer tissue (**A**) and many of these cells harboured segmented nuclei (**B**). Transforming growth factor-*β*-positive segmented nuclei cells were likewise observed in gastric (**C**, **D**) and colon (**E**, **F**) cancers, especially in the tumour periphery. Low power field (**A**, **C**, **E**). High power field (**B**, **D**, **F**).

CD68+ macrophages and antigranulocyte antibody-positive granulocyte cells were also distributed in the area surrounding the cancer nests, similar to the distribution of TGF-*β*-positive cells (Figure 5

). High-power field observation of these CD68+ macrophages had a single nucleus, while the antigranulocyte antibody-positive granulocyte cells harboured segmented nuclei. Thus, the cells that overexpress TGF-*β* can be confidently identified as granulocytes. Moreover, in gastric and colon cancer tissues, isolated cells with segmented nuclei around cancer nests at the invasive front also showed strong staining for TGF-*β* (Figure 6

).

### Double immunofluorescence stain, (TGF-*β*+CD68) or (TGF-*β*+granulocyte)

**Figure 7:**
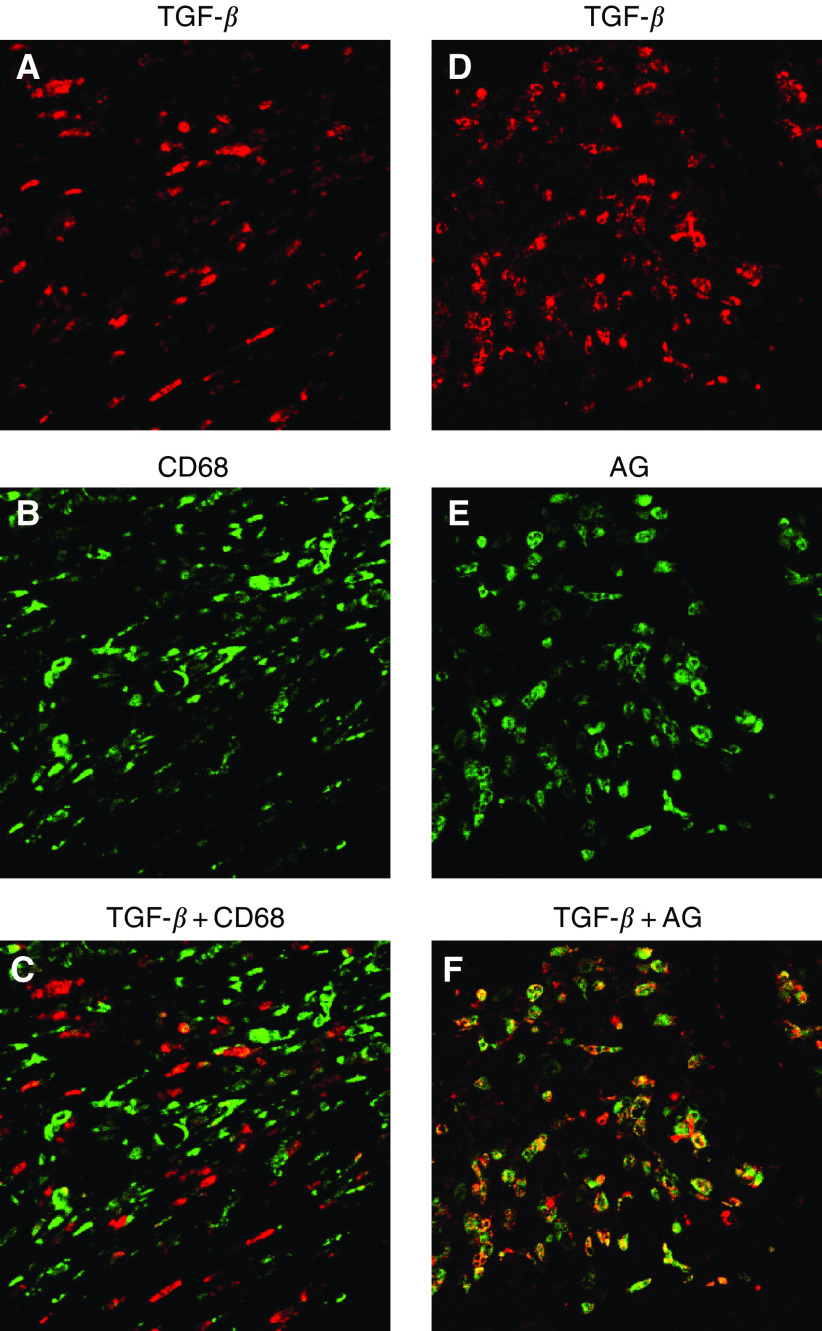
Confocal immunofluorescence images showing TGF-*β* (red) (**A**, **D**), CD68 as a marker of macrophages (green) (**B**) and granulocytes (green) (**E**). Transforming growth factor-*β* staining was topographically different from the staining of CD68+ cells (**C**). However, double staining with anti-TGF-*β* and antigranulocyte antibodies resulted in a consistent overlap (**F**). AG: antigranulocyte.

In order to unambiguously identify the cells producing TGF-*β* in pancreatic cancer tissues, double immunofluorescence staining was carried out. Distribution of TGF-*β*-positive cells did not correlate with the distribution of CD68+ cells (Figures 7A–C

). However, double staining with anti-TGF-*β* and antigranulocyte antibodies showed clearly concordant results (Figures 7D–F). These results indicated that the major cellular source of TGF-*β* in pancreatic tumour tissues, in addition to cancer cells, is granulocytes and not macrophages.

### Subtype of granulocytes by morphological observation

**Figure 8:**
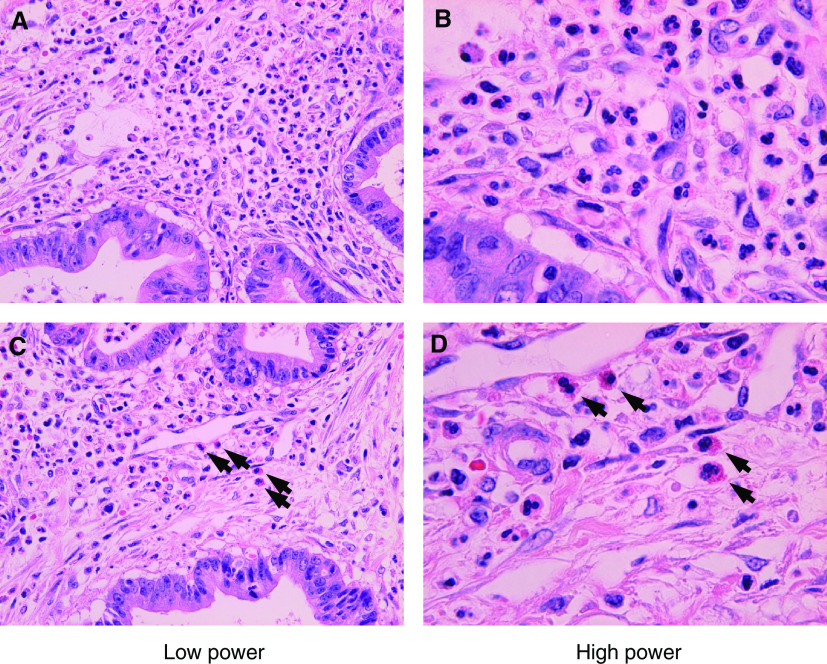
Morphological demonstration of infiltrated granulocytes in pancreatic cancer in Haematoxylin–eosin section. (**A**, **B**) Majorities of isolated cells in stromal, that was the precipitated area of TGF-*β*-staining positive cells, harboured segmented or polymorpho nuclei with neutrally stained cytoplasmic granules, indicating their neutrophilic nature. (**C**, **D**) Bilobed or trinuclei cells with acidic stained granules (=red), characteristic presentation of eosinophils, are also observed, but the proportion of these cells was at most 3 – 5% of the entire granulocyte. Low power field (**A**, **C**). High power field (**B**, **D**).

In order to identify the subclass of TGF-*β*-producing granulocytes, careful microscopical observation for haematoxylin–eosin-staining sections was performed. Majorities of the granulocytes were presumed to be neutrophils. As shown in representative pictures in Figures 8A and B

, these cells contain polymorphonucleus with cytoplasms stained pink, indicating their neutrophilic feature. Eosinophils, characterised by its distinctive cytoplasmic granules stained red (arrows in Figures 8C and D), were also noted, however, the population of this subtype should at most be 3 – 5% of entire granulocytes.

## DISCUSSION

Comprehensive mRNA analysis of several growth factors in pancreatic cancer, including TGF-*β*, CTGF, aFGF, bFGF, PDGF A, PDGF C and EGF, resulted in the finding that TGF-*β* is likely to be a potent inducer of the desmoplastic reaction. Furthermore, infiltrated granulocytes (mostly are neutrophils) were highlighted as a predominant source of TGF-*β*.

In this study, we initially demonstrated that the expression of type I and type III collagens in pancreatic cancer tissues was at least three-fold higher than that in normal pancreatic tissue (Figure 1A), confirming previous reports (Gress *et al*, 1995; Imamura *et al*, 1995; Linder *et al*, 2001). The origin of type I and type III collagens mRNA has been assumed to be cancer stroma (Gress *et al*, 1995), a finding supported by our studies in pancreatic cancer cell lines. Indeed, cancer cells are capable of synthesis and production of extracellular matrix proteins (Niitsu *et al*, 1988; Yoshida *et al*, 1989; Lohr *et al*, 1994), although the expression of collagen mRNAs in the pancreatic cancer cell lines was somewhat lower in comparison to that of fibroblast cell lines (Figure 3A). Furthermore, the expression of collagens from pancreatic cancer cells does not increase *in vivo*, that is, subcutaneous xenotransplantation in immunodeficient mice (unpublished data).

In order to better understand the molecular mechanisms underlying the stromal reaction, we examined the levels of several growth factors capable of inducing desmoplasia. Though plural molecules including TGF-*β*, aFGF, bFGF and PDGF C were overexpressed in pancreatic cancer (Figure 1B) and presumed to be associated with desmoplasia, we focused on TGF-*β* since its expression showed the most significant correlation with that of collagens. The expression of TGF-*β* in pancreatic cancer tissue was actually 3.5-fold higher than that found in normal pancreatic regions (Figure 1B). These results correlate with that of Friess *et al* (1993), who previously reported similar semiquantitative results by Northern blot analysis and/or *in situ* hybridisation. Other authors have reported on the overexpression of TGF-*β* in various cancer types by immunohistochemistry, Northern blot analysis and/or *in situ* hybridisation (Samuels *et al*, 1989; Yoshida *et al*, 1989; Barrett-Lee *et al*, 1990; Gorsch *et al*, 1992; Walker and Dearing, 1992; Mahara *et al*, 1994), although none of these methods were quantitative. Our results extend these earlier studies by providing independent, quantitative analysis of the overexpression of TGF-*β* in pancreatic cancer by employing real-time RT–PCR methods.

While the expressions of both TGF-*β* and collagens have been examined independently, their expressions relative to one another have not been considered. The utilisation of quantitative RT–PCR method enabled us to evaluate the correlation between the expressions of TGF-*β* mRNA and collagens. Both *in vitro* and *in vivo* experimental evidence has been accumulating, showing that TGF-*β* stimulates the production of collagens from fibroblasts. In fact, cultured fibroblasts increased the production of collagen from three- to five-fold when incubated with appropriate concentrations of TGF-*β* (Raghow *et al*, 1987; Varga *et al*, 1987). When TGF-*β* was directly injected into the subcutaneous tissue of newborn mice, accelerated fibrosis, that is, activation of fibroblasts to produce collagens, was demonstrated (Roberts *et al*, 1986; Shinozaki *et al*, 1997). In clinical diseases including pulmonary fibrosis and chronic renal allograft damage, TGF-*β* is also considered to be a main pathogenic factor for the overproduction of collagen (Nicholson *et al*, 1999). Obviously, TGF-*β* is not the only factor that can stimulate collagen expression in fibroblasts, since insulin and/or growth factors analysed here also regulate the production of type I collagen (Krupsky *et al*, 1996). Nonetheless, it can be stated here that TGF-*β* may be one of the main inducers of the desmoplastic reaction in pancreatic cancer.

One question concerns the cellular origin of TGF-*β* in pancreatic cancer nodules. Previous reports have indicated that the upregulated TGF-*β* originated from cancer cells, since immunohistochemical and/or *in situ* hybridisation studies demonstrated that the TGF-*β*s were localised in tumour cytoplasm (Friess *et al*, 1993; Coppola *et al*, 1998). However, it must be considered that the proportion of cancer cells in pancreatic cancer nodules is rather low (20 – 30% at the highest) as a result of desmoplastic reaction (Kloppel *et al*, 1985). If indeed these cancer cells were the origin of overexpressed TGF-*β* in pancreatic cancer nodules, each cancer cell would be expected to show prominent TGF-*β* staining. However, our immunohistochemical study for TGF-*β* demonstrated only faint cytoplasmic staining in cancer cells even after a 10-min reaction with DAB (Figure 4D). In contrast, isolated cells in the surrounding stroma of the cancer nests showed prominent positive staining even after short (1 min) DAB reaction times (Figure 4A). Initially, we assumed that this staining might be due to artefactual staining by endogenous peroxidase. However, staining without incubation of primary antibodies resulted in negative staining for these cells. A second possibility for false-positive staining could also be nonspecific binding of the Fc fragment or trapping of antibody in these isolated cells. In order to rule out this possibility, we carried out incubation with the first antibody with the same type of antibody against an antigen that is not expressed in human tissues, that is, polyclonal rabbit antibody against anti-GFP. Again, this negative control resulted in no staining in these isolated cells (Figures 4E and F). We are therefore confident that the isolated cells in stroma surrounding the cancer nest are actually strongly positive for TGF-*β*.

It then remained to determine the identity of the cells that overexpress TGF-*β*. We initially postulated that these cells were macrophages, since it has been reported that macrophages can secrete TGF-*β* (Assoian *et al*, 1987; Appleton *et al*, 1993) and, moreover, that the expression of TGF-*β* is associated with fibroblast collagen synthesis (Khalil *et al*, 1989). However, while the distribution of TGF-*β*-positive cells and CD68+ cells was somewhat similar, double-staining IHC did not show consistent double staining with these two antibodies (Figure 6). We then focused on granulocytes, since these cells, including neutrophils and eosinophils, have also been reported to express TGF-*β* (Grotendorst *et al*, 1989; Wong *et al*, 1991). Moreover, in the acute phase of disease, neutrophil granulocytes have been shown to express higher amounts of TGF-*β* mRNA with respect to lymphocytes and monocytes/macrophages (Ossege *et al*, 1996). Furthermore, the TGF-*β* produced by eosinophils has been shown to be involved in connective tissue remodelling and collagen synthesis (Stahle-Backdahl *et al*, 2000; Nomura *et al*, 2002). As shown in Figure 7, these TGF-*β*-positive cells coincided extremely well with that of antigranulocyte antibody-positive cells, and morphological evidence supported a notion that majorities of these cells were neutrophils (Figure 8). Together with previous reports that TGF-*β* is distributed in stromal inflammatory cells including granulocytes as well as cancer cells (Roberts *et al*, 1986; Assoian *et al*, 1987), it seems reasonable to regard that the predominant source of high levels of TGF-*β* may be infiltrating neutrophil, though bulk tumoral TGF-*β* should be accumulation of that from neutrophils, eosinophils and cancer cells. A precise and conclusive cellular source of TGF-*β* in a tumoral context, however, remains to be identified through *in situ* hybridisation.

Neutrophil infiltration is a biological phenomenon that is usually associated with acute inflammation such as bacterial infection. The present pancreatic cancer population was basically free from sign of acute pancreatitis showing high serum amylase level at the time of operation. Furthermore, resected specimens demonstrated no sign of infection such as the presence of pus. We believe that this neutrophil infiltration observed in the present study may be an important phenomenon that should be focused in understanding pancreatic cancer progression. Observation of only the central core of the pancreatic cancer may have missed this neutrophil infiltration, as we demonstrated in Figure 4. In order to evaluate whether infiltration of granulocytes overexpressing TGF-*β* is specific to pancreatic cancer, we performed immunostaining for TGF-*β* on gastric and colon cancer samples. As shown in Figure 6, TGF-*β*-positive granulocytes are clearly present, especially at the invasive front. Thus, TGF-*β*-secreting infiltrating granulocytes are present in pancreatic, gastric and colon cancer; however, a prominent desmoplastic reaction is observed only in the former. This apparent discrepancy may be explained by the mechanism of activation of TGF-*β*. Transforming growth factor-*β* is generally released from cells in a latent, biologically inactive form (Miyazono *et al*, 1993). Following release, it is activated by a variety of mechanisms, including exposure to proteolytic enzymes or alkaline pH conditions (Gleizes *et al*, 1997; Khalil, 1999). The presence of proteases in the pancreas and the alkaline pH of pancreatic juice may result in ideal conditions for activation of latent TGF-*β*.

In conclusion, we demonstrated that TGF-*β* is overexpressed in pancreatic cancer nodules and, moreover, that TGF-*β* is secreted mainly by infiltrating granulocytes (mostly are neutrophils) and not cancer cells. Once secreted, TGF-*β* can be activated in the unique pancreatic environment, thereby stimulating fibroblasts to produce collagens. In order to interfere with this desmoplastic reaction in pancreatic cancer, a greater understanding and control of the phenomenon of granulocyte infiltration, and control of subsequent activation mechanisms of TGF-*β*, is urgently required. Furthermore, the meaning of neutrophils infiltration in pancreatic cancer progression, that is, whether it is associated with better or worse prognosis, remains to be elucidated.
